# Synergistic Thermal and Electron Wind Force-Assisted Annealing for Extremely High-Density Defect Mitigation

**DOI:** 10.3390/ma17133188

**Published:** 2024-06-29

**Authors:** Md Hafijur Rahman, Sarah Todaro, Daudi Waryoba, Aman Haque

**Affiliations:** 1 Department of Mechanical Engineering, The Pennsylvania State University, University Park, PA 16803, USA; mxr5923@psu.edu (M.H.R.); set5410@psu.edu (S.T.); 2 Engineering, Applied Materials, Penn State University, 1 College Place, DuBois, PA 15801, USA; drw29@psu.edu

**Keywords:** defect mitigation, cold rolling of FeCrAl alloy, electron wind force (EWF), electron backscatter diffraction (EBSD), X-ray diffraction (XRD)

## Abstract

This study investigates the effectiveness of combined thermal and athermal stimuli in mitigating the extremely high-density nature of dislocation networks in the form of low-angle grain boundaries in FeCrAl alloy. Electron wind force, generated from very low duty cycle and high current density pulses, was used as the athermal stimulus. The electron wind force stimulus alone was unable to remove the residual stress (80% low-angle grain boundaries) due to cold rolling to 25% thickness reduction. When the duty cycle was increased to allow average temperature of 100 °C, the specimen could be effectively annealed in 1 min at a current density of 3300 A/mm^2^. In comparison, conventional thermal annealing requires at least 750 °C and 1.5 h. For specimens with 50% thickness reduction (85% low-angle grain boundaries), the electron wind force was again unable to anneal the defects even at 3300 A/mm^2^ current density and average temperature of 100 °C. Intriguingly, allowing average concurrent temperature of 200 °C eliminated almost all the low-angle grain boundaries at a current density of 700 A/mm^2^, even lower than that required for the 25% thickness reduced specimens. Comprehensive electron and X-ray diffraction evidence show that alloys with extremely high defect density can be effectively annealed in less than a minute at approximately 200 °C, offering a substantial improvement over conventional high-temperature annealing.

## 1. Introduction

Residual stress is a critical factor in the design, manufacturing, and reliability of engineering components and structures [1,2,3]. For example, cold rolling is widely used to produce thin metallic sheets and strips for applications in numerous industries, including automotive/aerospace, construction/packaging, and energy. The process injects a large amount of residual stress into the materials by introducing very high-density dislocations, also known as low-angle grain boundaries (LAGBs) [4,5]. Welding is another prevalent technique in manufacturing that generates extremely high residual stress in the joint of two metallic components. Very high dislocation density increases the strength but may also decrease toughness, since these terms are generally mutually exclusive [6]. Effective control of the dislocation density is therefore crucial to achieve an acceptable combination of strength and toughness, for which heat treatment has been the only technique available in the literature. Depending on the alloying elements and the residual stress (or LAGB density), heat treatment may require very high temperatures to activate dislocation movement and recrystallization processes [7]. Potential risk of unwanted phase transformation is present due to the high processing temperature, which can compromise materials’ properties [8,9]. Moreover, achieving uniform temperature distribution during annealing can be challenging for complex geometries, leading to inconsistent treatment results. Since thermal diffusion is essentially random, the conventional annealing process takes a long time.

While thermal annealing is practically the only technique available for microstructural control, the literature is growing on electrical annealing with pulsed current [10,11,12]. Pulsed current may generate both thermal (Joule heating) and athermal (electron wind force, or EWF) stimuli. Joule heating originates from the collisions of the drifting electrons with lattice atoms. The EWF, on the other hand, is generated only when the electrons collide with a defect and loses its momentum due to the process [13]. The transferred momentum is the origin of the EWF, which is therefore highly specific to defects. The EWF is purely mechanical in nature and can potentially mobilize and/or anneal defects, such as dislocations and grain boundaries [14,15]. The EWF is particularly beneficial for defect mitigation as it does not involve heating. More importantly, the EWF is highly directional in nature [16], which is in stark contrast with thermal annealing. The combination of defect specificity and directional diffusion attributes of the EWF may enable the ultrafast annealing effect at room temperature. However, it is argued whether EWF alone is potent enough to activate a dislocation motion. The literature indicates that both high current density (to increase the EWF magnitude) and high temperature (to increase atomic mobility) are needed for defects to migrate and be annihilated. As a result, electrical pulse annealing has predominantly included Joule heating to raise the temperature [10,12,15,17].

The core theme of this study is the potential synergy of the EWF and Joule heating in reducing residual stress. The motivation is to explore the current–temperature–materials topology to see if the total amount of energy can be reduced for the same annealing effect. We argue that for very high initial LAGB density, the EWF or temperature may never be potent enough to disentangle the dislocation network if acting separately. However, when applied simultaneously, relatively lower temperature may induce enough atomic vibration in the non-equilibrium (defects) atoms for the low magnitude EWF to push them beyond cutoff interatomic distance. This is feasible only because EWF is highly specific to defects (lower temperature will not affect atoms at equilibrium distance, which are deep into the interatomic potential well). To study this, we developed an experimental technique where extremely small (20 microsecond pulse width) and sharply defined current pulses are passed through the specimens. Such tightly controlled pulses allow us to tune the temperature effectively. For example, frequency as low as 2 Hz essentially keeps the average temperature close to the ambient. Raising the frequency can controllably raise the temperature. Therefore, the setup can decouple the temperature and EWF effects, which is necessary to investigate their synergy.

In this study, we demonstrate the synergy of temperature and the EWF on FeCrAl alloy, which has excellent high temperature and oxidation resistance [18,19]. The addition of aluminum not only enhances the oxidation resistance by forming a stable Al_2_O_3_ surface scale but also contributes to the alloy’s overall high-temperature performance [19,20]. Similarly, chromium plays a crucial role in stabilizing this protective oxide layer, extending the material’s service life in corrosive environments [21]. These characteristics make FeCrAl alloys highly valuable in sectors such as aerospace and power generation, where materials are subjected to extreme conditions. Notably, these alloys are increasingly recognized for their potential in nuclear applications, specifically as cladding materials in light-water reactors, where their superior resistance to high-temperature steam and water vapor significantly enhances safety and reliability [22,23]. Cold rolling is a widely used method for fabricating cladding tubes [24]. The resulting residual stress may adversely affect the alloy’s performance under operational conditions [25,26]. For instance, the increased dislocation density can facilitate crack initiation under stress, potentially leading to premature material failure [27]. Moreover, the texture imparted by cold rolling can affect the material’s corrosion resistance and physical properties in anisotropic ways [28,29]. The influence of EWF-assisted annealing on the mechanical and structural properties of FeCrAl alloys is complex and dependent on the specific alloy composition. Preliminary investigations into this treatment method have revealed subtle yet significant changes in material characteristics. For instance, it has been observed that the hardness of FeCrAl alloys can slightly increase as a result of EWF-assisted annealing [30]. This enhancement in hardness is attributed to the interaction between the electron wind force and the material’s microstructure, promoting defect healing and inducing microstructural changes that strengthen the alloy.

Our hypothesis posits that when acting together, thermal and athermal stimuli can enhance the mobility and annihilation of dislocations with higher efficiency compared to when acting separately. The implication is the possibility of annealing high-temperature alloys such as the FeCrAl at temperatures well below the typical recrystallization threshold, particularly in samples with very high dislocation densities. To validate this hypothesis, we rolled FeCrAl alloy specimens to reduce the thickness by 25% and 50% and then investigated the synergy of applied EWF and temperature. The maximum current density for the EWF was limited to 3300 A/mm^2^ to avoid possibilities of electromigration failure [31,32]. The average temperature was also limited to 200 °C, which is significantly lower than the annealing temperature reported in the literature. We performed detailed microstructural analyses using electron backscatter diffraction (EBSD) and X-ray diffraction (XRD), focusing on parameters such as low-angle grain boundaries (LAGBs), kernel average misorientation (KAM), and the full width at half maximum (FWHM) of the XRD peaks. We also compared the outcomes of EWF-assisted annealing with conventional thermal annealing at 200 °C, which failed to mitigate any defects, thus demonstrating the superior efficacy of the combined thermal and athermal approach in defect reduction within FeCrAl alloys.

## 2. Materials and Methods

**Figure 1:**
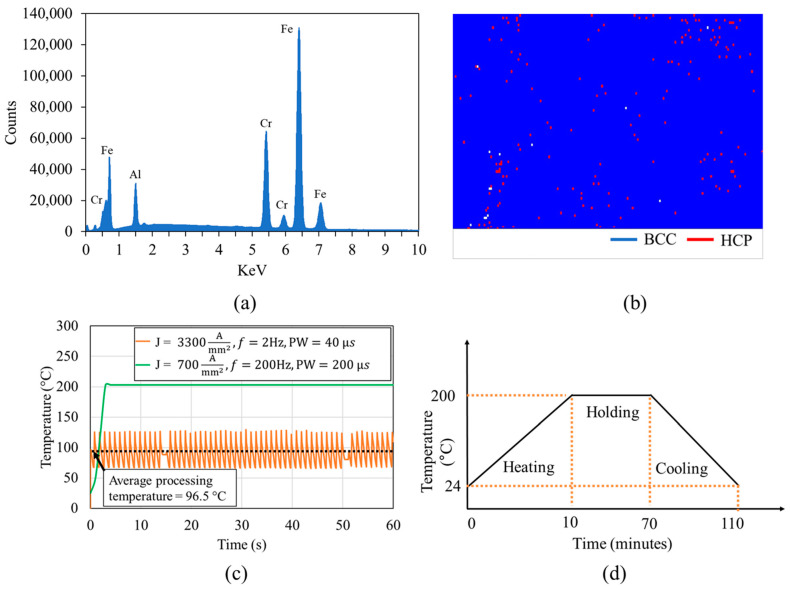
(**a**) Energy dispersive X-ray (EDX) spectrum of materials; (**b**) EBSD phase map of alloy (white dots are points of zero solution); (**c**,**d**) temperature profile during (**c**) various EWF-assisted annealing and (**d**) thermal annealing for benchmarking purpose.

In this study, we utilized FeCrAl alloy samples, which were initially confirmed for composition (Fe-73%, Cr-21.3%, Al-5.7%) via Energy Dispersive X-ray (EDX) analysis. The phase distribution of the alloy was determined through Electron Backscatter Diffraction (EBSD). The EBSD phase map showed that the alloy comprises predominantly the BCC phase, accounting for approximately 98%, with the remainder being the HCP phase (Figure 1). The FeCrAl alloy samples were subjected to cold rolling at room temperature using a manual hand-operated rolling mill. The alloy underwent two distinct phases of cold rolling, initially to a ~25% thickness reduction, and subsequently to a more severe ~50% reduction, to systematically increase the defect densities. Each pass during rolling was calibrated to ensure uniform reduction across the entire width of the sample. Lubrication was applied to minimize friction and heat generation, which could affect the microstructure. The electrical processing was conducted after each step of rolling using a programmable DC power supply (Magna-power, SL600-2.5/UI, Flemington, NJ USA) paired with a current pulse generator (Eagle Harbor Technologies, Inc., IPM-16P-2003, Seattle, WA, USA). To precisely monitor temperature changes during the treatment, a thermal microscope (Optris Infrared Sensing, LLC, Optris PI 640, Portsmouth, NH USA) was employed. The effective average temperature was controlled by current pulse width and frequency. Figure 1a shows the individual control of the current density and temperature. Here, very high current density (3300 A/mm^2^) with 40 μs pulse width and 2 Hz frequency result in average temperature below 100 °C, whereas only 700 A/mm^2^ current density can lead to about 200 °C for 200 μs pulse width and 200 Hz frequency, respectively. It is to be noted that we tailored the processing parameters based on our prior experimental insights [30]. Initially, for the sample with lower defect density (25% thickness reduced), we minimized frequency and pulse width (2 Hz and 40 µs, respectively) to predominantly utilize the electron wind force (EWF). We incrementally increased the current density while continuously monitoring changes in electrical resistance. At a current density of 3300 A/mm^2^, a notable change in resistance was observed which indicated significant microstructural modification. For the 50% rolled samples, characterized by a higher defect density, the previous settings were found insufficient. Thus, we increased the frequency and pulse width to enhance the thermal contribution, critical for supporting defect resolution under higher deformation. Given the elevated temperatures, to mitigate the risk of electromigration, we maintained a lower current density of 700 A/mm^2^. Adjustments were made until the processing temperature reached 200 °C, at which point significant resistance changes were observed again at settings of 200 Hz and 200 µs. It is important to note that the materials’ parameters (alloying elements and initial defect density) strongly influence these outcomes. Since we were able to anneal all the LAGBs at 200 °C and 700 A/mm^2^, we performed conventional annealing at 200 °C alone to highlight the synergy with the EWF. This involved heating at 0.3 °C/s for 10 min, maintaining the temperature at 200 °C for one hour, and cooling at 0.075 °C/s for 40 min (Figure 1b).

A comprehensive characterization was performed using the electron backscatter diffraction (EBSD) to evaluate microstructure evolution and defect densities. Before performing the EBSD analysis, the sample was subjected to detailed surface preparation to ensure its suitability for microscopic examination. This preparation included a polishing process using a rotary tool and progressively finer diamond compounds, from 3000 to 120,000 grit. Following this, ion milling was conducted at 4.5 kV and 1.5 A for 90 min to refine the surface further, ensuring optimal conditions for the generation of the Kikuchi line pattern that is necessary for the EBSD analysis. EBSD analysis was carried out with a VERIOS G4 UC Scanning Electron Microscope (Thermo Scientific, Hillsboro, OR, USA), operating at a beam current of 3.2 nA, an accelerating voltage of 20 KV, and a step size of 0.3 μm. Data collection was facilitated by the Aztec software suite version 6.2 and the collected EBSD data were then post-processed and analyzed using the Aztec Crystal software suite version 5.1 [33]. The EBSD results were complemented by X-ray diffraction (XRD) to monitor shifts in peak broadening as indicators of residual stress and defects. X-ray diffraction was performed using a Malvern Panalytical Empyrean^®^ diffractometer equipped with a cobalt line-focus X-ray tube, which has a wavelength of 1.7889 Å (Malvern, UK). The instrument was operated at a voltage of 40 kV and a current of 40 mA. Measurements were taken over a 2θ range from 35° to 125° with a step size of 0.016°. The diffractometer was set with a divergence slit of ${\left(\frac{1}{16}\right)}^{{}^{\circ}}$, a mask of 2 mm, a soler slit of 0.04 rad, and an anti-scatter slit of ${\left(\frac{1}{4}\right)}^{{}^{\circ}}$, optimizing the setup for comprehensive sample area irradiation.

## 3. Results

**Figure 2:**
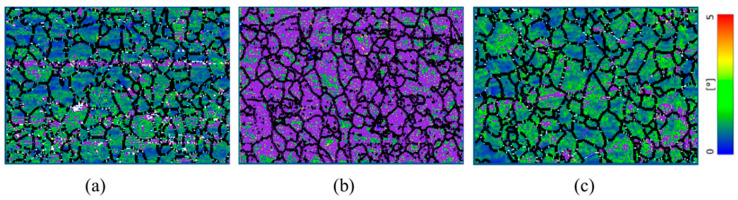
Grain boundaries and kernel average misorientation maps for (**a**) pristine, (**b**) ~25% rolled and (**c**) EWF-annealed at ~100 °C. The color scale from 0° to 5° represents KAM values, with violet indicating low-angle grain boundaries (LAGBs) defined within the range of 2° ≤ θ ≤ 10°, and black color indicates high angle grain boundaries (HAGBs) (θ > 10°).

The dislocations generated during cold rolling are usually accommodated within the grains as low-angle grain boundaries (LAGBs) as evidenced in Figure 2. Initially, in its pristine state, the alloy exhibited a relatively low LAGB concentration, ~54.8%. However, after the first phase of rolling, which involved a 25% reduction in thickness, the LAGB concentration increased to ~80%. Mitigating these entangled dislocations is essential for improving material properties across various applications. The defects induced by ~25% cold rolling was effectively mitigated at a current density of approximately $3300\;\frac{A}{mm^{2}}$, a pulse width of 40 microseconds, and a frequency of 2 Hz [30].

### 3.1. Synergistic Thermal and Athermal Effects in Dislocation Density Mitigation

In metallurgical processes, the effectiveness of defect-mitigation techniques, particularly electropulsing, is critically dependent on the initial microstructural state of the material, influenced by processes such as cold rolling. Usually, the electron wind force is conjectured to rapidly disrupt the entangled dislocations that hinder dislocation mobility [14]. However, in cases of extensive deformation, such as with 50% cold rolling, dislocation networks become more intricate and tightly interwoven. The thermal component of the annealing process may reduce the energy barriers for such complex entanglements, which effectively facilitates the subsequent electron wind force action. This reduction in energy barriers softens the rigidity of the dislocation networks, allowing the electron wind force to more efficiently promote dislocation migration. Concurrently, this thermal component of the treatment enhances atomic diffusion, aiding in the migration of dislocations towards and along grain boundaries. Consequently, the combined action of EWF and the thermal force then drives dislocations toward high-angle grain boundaries, which act as effective sinks for these defects, facilitating their annihilation. In addition, this process potentially leads to the formation of dislocation walls, which can transform low-angle grain boundaries (LAGBs) into high-angle grain boundaries (HAGBs) [30]. This synergistic effect between thermal and athermal energies thus results in a more thorough and efficient reconfiguration of the microstructure, thereby reducing residual stresses more effectively than either treatment could alone.

**Figure 3:**
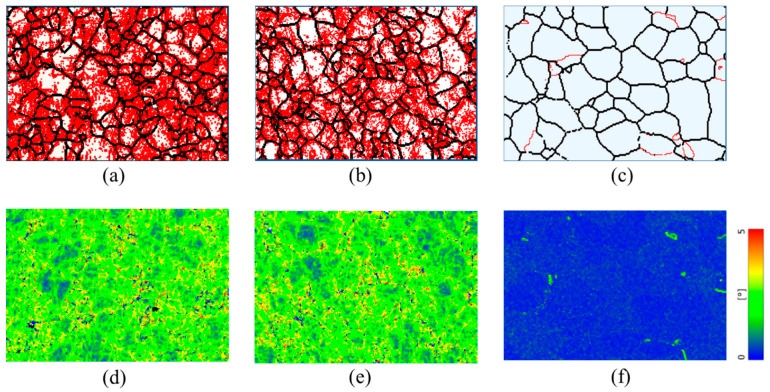
Grain boundaries (**a**–**c**) and kernel average misorientation (KAM) maps (**d**–**f**) of (**a**,**d**) ~50% rolled; (**b**,**e**) EWF annealed at ~100 °C with current density of $3300\;\frac{A}{mm^{2}}$; (**c**,**f**) EWF annealed at ~200 °C with current density of $700\;\frac{A}{mm^{2}}.$ Red color in panels (**a**–**c**) indicates low-angle grain boundaries (LAGBs) (2° ≤ θ ≤ 10°) and the black color indicates the HAGBs (10° < θ ≤ 100°). The color scale in panels (**d**–**f**) displays the misorientation angles from 0° to 5°.

In this experiment, during an initial setup where the material underwent 25% thickness reduction through cold rolling, applying electropulsing at a current density of $3300\;\frac{A}{mm^{2}}$ at approximately 100 °C was found sufficient to effectively mitigate these defects close to its pristine state (Figure 2c), as described in our previous publication [30]. At this moderate level of deformation, the dislocation density—while increased—remained manageable, allowing the applied electropulsing treatment to mobilize and reduce these dislocations efficiently. However, when the experiment was scaled to a more severe condition with 50% rolling reduction, the same electropulsing parameters did not yield comparable results. At 50% rolling reduction, despite the low-angle grain boundary (LAGB) concentration appearing similar (~85%) to the levels observed at 25% rolling (Figure 2b and Figure 3a), the dislocation networks formed are considerably more complex and entangled. The reason is, other forms of defects, particularly vacancy clusters, are likely more pronounced due to the higher degree of deformation [34]. Čížek et al. [34] have demonstrated that higher levels of deformation in materials not only introduce a proliferation of dislocations but also generate a high concentration of vacancies. A significant portion of these deformation-induced vacancies dissipate by diffusing to sinks such as grain boundaries and dislocations. However, the remaining vacancies tend to agglomerate, forming larger vacancy clusters whose average size increases with the degree of deformation. In addition, cold rolling reduces the distance between dislocations due to an increase in dislocation density, as reported in [35,36]. Although cold rolling accelerates solute atom diffusion [37], which could potentially facilitate some aspects of microstructural recovery, the combination of dense, tangled dislocation networks and enlarged vacancy clusters may complicate the microstructure. This complexity may hinder the overall mobility of dislocations, posing challenges to their effective rearrangement and annihilation under electropulsing treatment, thereby impacting the efficiency of defect mitigation in these heavily deformed alloys. Observations from Figure 3b highlighted that the defects remained largely unmitigated under the similar electropulsing settings (current density $3300\;\frac{A}{mm^{2}}$, average processing temperature ~100 °C). It is to be noted that EBSD, the technique employed in our analysis, does not have the capability to detect vacancy defects, which may also play a significant role in the material’s behavior post-deformation (such as 25% or 50% rolled).

In addition to low-angle grain boundary (LAGB) concentration, we examined the Kernel Average Misorientation (KAM) maps to assess the internal strain and dislocation density changes due to cold rolling and subsequent annealing treatments. The pristine condition KAM map of this sample is discussed in our previous study [30]. The increase in KAM values after 50% rolling clearly indicates a rise in internal strain, which corresponds to the increased dislocation density and the resultant residual stress within the material, as noted in previous studies [38]. Li et al. [39] also corroborated that deformed grains typically show elevated KAM values due to the higher dislocation densities. From the KAM maps, we observed that the Electron Wind Force (EWF) annealing at 100 °C with a current density of $3300\;\frac{A}{mm^{2}}$ did not significantly alter the KAM values for the 50% rolled sample. This further suggests that the applied treatment parameters were insufficient to effectively mitigate the more severe dislocations and residual stresses introduced by this higher degree of deformation. In contrast, for the 25% rolled sample, our previous publication [30] documented a complete restoration of the KAM values under the same EWF annealing conditions, indicating effective stress and defect mitigation at this lower level of deformation. This differential response highlights the critical role of initial defect severity and suggests that adjustment of treatment parameters might be necessary to achieve similar mitigative effects in more heavily deformed materials.

**Figure 4:**
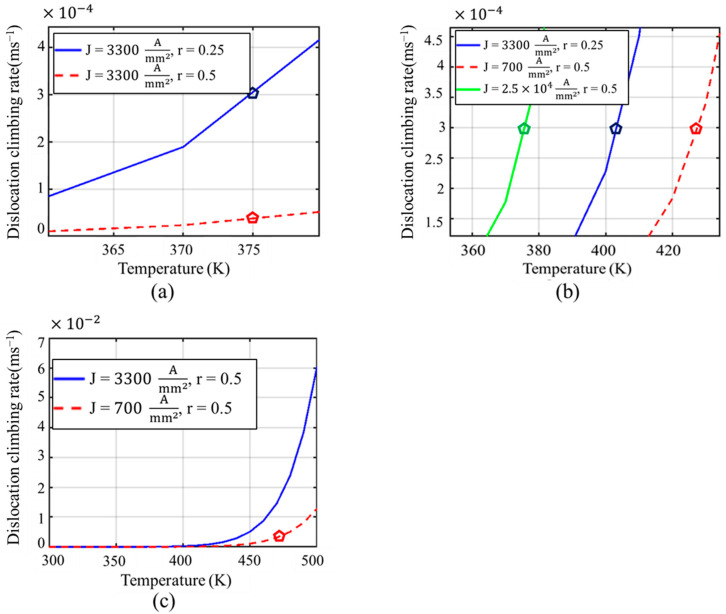
(**a**–**c**) Dislocation climbing rate for various annealing conditions (J is the current density, r = 0.25 and 0.5 represent 25% and 50% rolling, respectively).

To elucidate the underlying mechanisms governing the reduction of dislocation density through electropulsing treatment, we developed a simplified mathematical model that accounts for both thermal and athermal contributions, as well as the effects of rolling-induced deformation. We modified the relation for the current-assisted dislocation climbing rate, $v=4\pi ac_{0}D_{v}\frac{fV_{a}}{lkT},$ as described in [40]. Here, *a* as the atomic transition interval, $c_{0}\;$ is the density of vacancy sites, $V_{a}\;$ is the atomic volume, and $\frac{f}{l}$ is the climbing force. We know that the electron wind force (EWF), generated by the momentum transfer from the pulsed current, exerts a mechanical shock on dislocations, facilitating their movement. This force, $F_{EWF}$, is given by $F_{EWF}=\frac{2h}{\pi }nj$ [41], where *h* is Planck’s constant, *n* is the electron density, and *J* is the current density. In our simplified model, we assume that the electron wind force is entirely utilized as the climbing force, $\frac{f}{l}.$ Concurrently, Joule heating raises the local temperature, enhancing atomic mobility and promoting dislocation climb. The temperature-dependent diffusion coefficient, $D_{v}$, follows an Arrhenius-type behavior, $D_{v}=D_{0}e^{-\frac{E_{a}}{KT}}$, where $D_{0}$ is the pre-exponential factor, $E_{a}$ is the activation energy, *k* is Boltzmann’s constant, and *T* is the absolute temperature. To incorporate the effect of rolling, we modified $D_{v}$ as $D_{v}r^{\alpha }$, where *r* is the rolling percentage (e.g., *r* = 0.5 for 50% rolling) and *α* is an experimental coefficient representing the influence of rolling on atomic mobility. Furthermore, the dislocation climbing rate *v* is adjusted by a factor of $e^{-r}$, accounting for the increased complexity and density of dislocation networks due to higher rolling percentages. The modified dislocation climb rate thus becomes $v=4\pi ac_{0}D_{v}r^{\alpha }(\frac{F_{EWF}V_{a}}{kT})e^{-r}$. A high dislocation climbing rate signifies that dislocations can move more quickly, leading to more efficient rearrangement and annihilation of these defects. For instance, at a fixed temperature, if one scenario has a higher climbing rate than another, the dislocations in the former can more rapidly migrate, interact, and be annihilated, resulting in a more significant reduction in dislocation density [40,42]. To explain the observed experimental phenomenon, this simplified model is solved in MATLAB (version 2022a) and the solutions for different scenarios are plotted in Figure 4.

From Figure 4a, we observed that the dislocation climbing rate, which indicates the ease with which dislocations traverse through the crystal lattice, was adequately high for 25% rolled sample, annealed at 100 °C with a current density of $3300\;\frac{A}{mm^{2}}$, to facilitate significant rearrangement and annihilation of dislocations. However, under the same annealing conditions, the dislocation climbing rate for the 50% rolled sample was markedly lower compared to the 25% rolled condition. This decreased climbing rate at the higher rolling condition directly impacts the efficacy of defect mitigation. With dislocations less able to climb and thus less mobile, their capacity to move out of their locked positions decreases, hampering the effectiveness of the electropulsing treatment. The reduced climbing rate observed in the 50% rolled sample implies that dislocations are not sufficiently mobilized to overcome the increased barriers presented by the more dense and complex dislocation networks. This explains our observations in Figure 3b,e, where both the LAGB and KAM values remain unchanged, which indicates a persistence of high dislocation densities despite the treatment. This suggests a need for either higher temperatures, increased current densities, or prolonged treatment durations to achieve similar levels of defect mitigation as seen with less severe deformations.

To achieve a dislocation climbing rate for the 50% rolled sample comparable to that effective for the 25% rolled sample at 100 °C, we explored two potential strategies. First, according to our mathematical model and as illustrated in Figure 4b, increasing the current density to $2.5\times 10^{4}\frac{A}{mm^{2}}$ could theoretically replicate the desired climbing rate at 100 °C for the 50% rolled sample. However, this approach was intentionally disregarded due to the high risk of electromigration failure associated with such a significant increase in current density at 100 °C. Electromigration, the transport of material caused by the momentum transfer from the moving electrons, can lead to significant material degradation and failure, particularly under this high current density at elevated temperature [31,32]. The second, more viable option involves allowing a controlled increase in temperature. Our analyses showed that at approximately 160 °C, a much lower current density of $700\;\frac{A}{mm^{2}}$ is sufficient to achieve a climbing rate similar to that of the 25% rolled sample treated at 100 °C with $3300\;\frac{A}{mm^{2}}$ (Figure 4b). This approach leverages the temperature’s influence on enhancing atomic mobility and dislocation dynamics without the severe risk of electromigration. From Figure 2a, significant defects were noted in the pristine sample, likely introduced during the fabrication process [43]. To address both the inherent defects from fabrication and those induced by 50% rolling, we opted to adjust our electropulsing treatment parameters further. Instead of maintaining the temperature at 160 °C, we allowed it to rise to 200 °C while maintaining a current density of $700\;\frac{A}{mm^{2}}$. This strategic adjustment was aimed not only at mitigating the defects associated with rolling but also at addressing the initial defects present in the material. Figure 4c provides insights into the effectiveness of this approach. At 200 °C and $700\;\frac{A}{mm^{2}}$ current density, the dislocation climbing rate was observed to be higher than that at 100 °C with a current density of $3300\;\frac{A}{mm^{2}}$, indicating more favorable conditions for dislocation movement and annihilation. The resultant microstructural analysis from Figure 3c further corroborated the success of this treatment, revealing that the low-angle grain boundary (LAGB) concentration decreased dramatically to only 10%—significantly lower than the ~54.8% observed in the pristine condition. Additionally, the KAM distribution also shows significant reduction, as illustrated in Figure 3f, further evidencing the successful mitigation of microstructural defects. This substantial reduction in LAGB concentration and KAM highlights the efficacy of increasing the treatment temperature to 200 °C, demonstrating that higher temperatures can facilitate more effective defect mitigation, even at lower current densities. This simplified model demonstrates that the combined application of EWF and moderate Joule heating can effectively mobilize and annihilate dislocations, even in heavily deformed materials, thereby mitigating dislocation density more efficiently than traditional thermal annealing alone. However, determining the precise value of the experimental coefficient α requires additional experimental datasets. The simulations based on this model align well with our experimental observations, reinforcing the synergistic impact of thermal and athermal stimuli in defect mitigation, especially in the context of high rolling percentages.

### 3.2. Thermal Annealing and EWF-Assisted Annealing Performance Comparison

**Figure 5:**
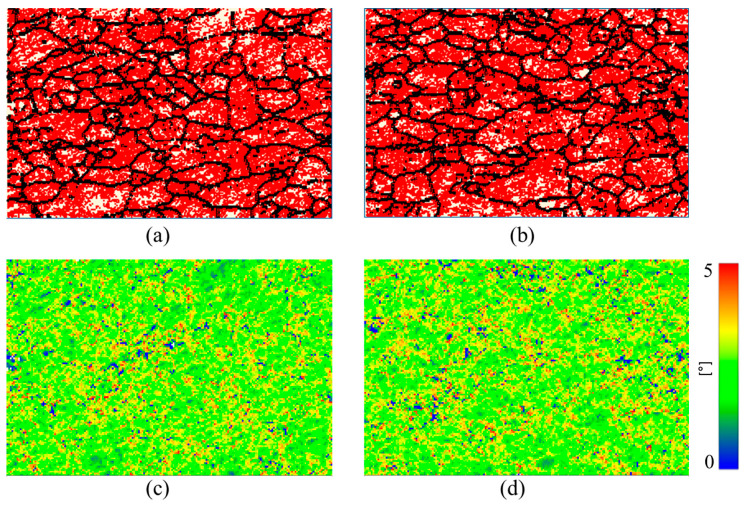
Grain boundaries (**a**,**b**) and kernel average misorientation (KAM) maps (**c**,**d**) of (**a**,**c**) ~50% rolled; (**b**,**d**) thermal annealed at ~200 °C for 110 min. In panel (**a**,**b**) Red color indicates low-angle grain boundaries (LAGBs) (2° ≤ θ ≤ 10°) and black color indicates high angle grain boundaries (HAGBs) (θ > 10°). The color scale in panels (**c**,**d**) displays the misorientation angles from 0° to 5°.

To compare the effectiveness of EWF-assisted annealing with conventional thermal annealing, thermal annealing was performed at the same temperature of 200 °C. The results of this thermal annealing were remarkably different from those achieved with EWF-assisted annealing at the same temperature. Notably, the concentration of low-angle grain boundaries (LAGBs) remained unchanged after thermal annealing as shown in Figure 5b. Furthermore, the Kernel Average Misorientation maps revealed that thermal annealing at 200 °C did not reduce the KAM values either (Figure 5d), indicating that this low temperature was inadequate to facilitate any substantial microstructural improvements. This lack of change can be attributed to the nature of thermal annealing, which primarily relies on thermal diffusion processes for defect mitigation. For thermal annealing to effectively reduce defects, the material typically needs to be treated above its recrystallization temperature. For FeCrAl, this temperature is around 700 °C [44,45]. At 200 °C, the energy provided is insufficient to activate the necessary diffusion processes that facilitate defect rearrangement and reduction. This highlights a significant limitation of traditional thermal annealing for materials like FeCrAl, where high processing temperatures are required to achieve noticeable microstructural improvements.

In contrast, as discussed in earlier section, the synergistic application of electron wind force (EWF) and thermal effects during EWF-assisted annealing at 200 °C demonstrated a remarkable ability to reduce both the LAGB concentration and KAM effectively (Figure 3). This suggests that the combination of EWF and moderate thermal input can enhance the defect mobility and interaction at much lower temperatures than those required for conventional thermal annealing. The EWF likely provides additional mechanical energy that aids in overcoming the activation barriers for dislocation movement, effectively enhancing the material’s response to the thermal component of the treatment.

To quantitatively assess the effectiveness of different annealing parameters on defect mitigation, Table 1 presents a comparative analysis of low-angle grain boundary (LAGB) concentrations observed across various experimental conditions.

### 3.3. X-ray Diffraction Analysis

**Figure 6:**
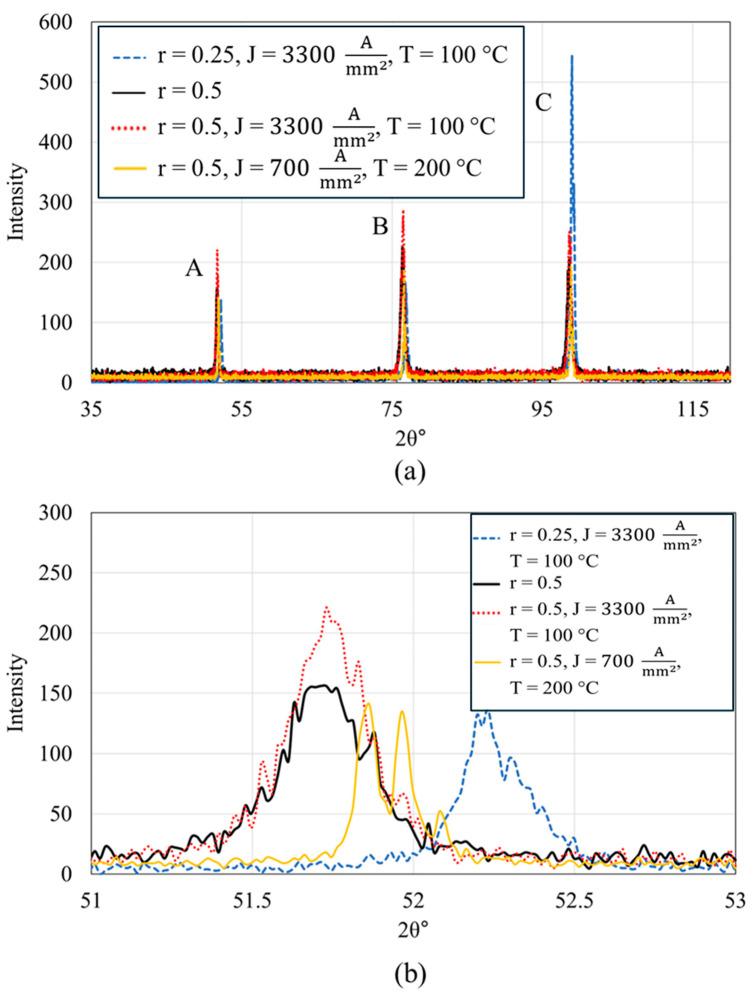

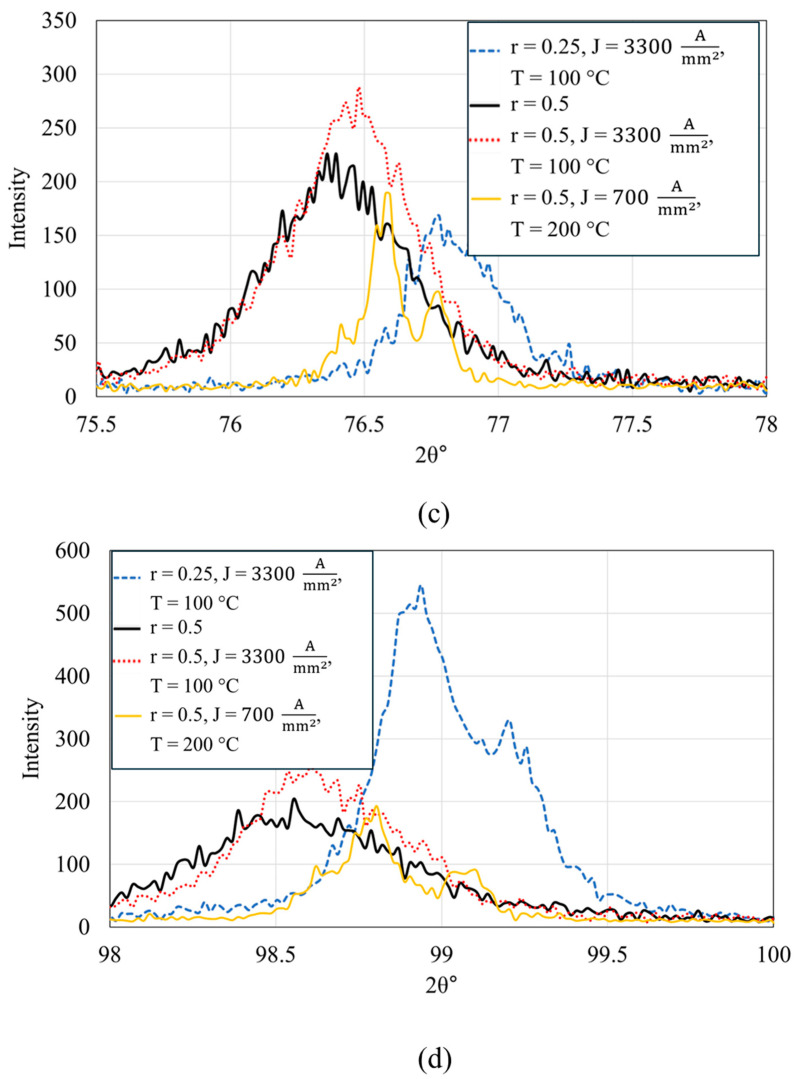
X-ray diffraction patterns of FeCrAl alloy under various conditions; (**a**) full spectrum from 35° to 125° showing three distinct peaks labeled Peak A, Peak B, and Peak C; (**b**–**d**) Zoomed-in views of Peak A, Peak B, and Peak C, respectively, highlighting variations due to changes in rolling percentage (r) and annealing parameters such as current density (J) and temperature.

The comprehensive X-ray diffraction (XRD) analysis details the microstructural changes in a FeCrAl alloy subjected to mechanical rolling and subsequent electron wind force (EWF)-assisted annealing. In the pristine FeCrAl sample, three major peaks are identified, denoted as Peak A, Peak B, and Peak C, as shown in Figure 6. The analyses of peak position shifts and changes in full width at half maximum (FWHM) induced by mechanical deformation and subsequent annealing processes are detailed in Table 2 and Table 3, respectively. Upon rolling to 25%, a subtle leftward shift in the peak positions to lower 2θ values and significant increase in the full width at half maximum (FWHM) are observed, which indicates the introduction of dislocations and residual stresses causing lattice strain [46]. In our previous research [30], we demonstrated that EWF-assisted annealing at 100 °C with a current density of $3300\;\frac{A}{mm^{2}}$ effectively reduced the FWHM close to the pristine value. However, when the rolling is intensified to 50%, the peak positions exhibit a more pronounced leftward shift (for instance, peak A shift ~0.53° leftward). Correspondingly, the FWHM of each peak increases more significantly for the 50% rolled sample, indicating a greater accumulation of internal stresses and defects [47,48]. After EWF-assisted annealing at a current density of $3300\;\frac{A}{mm^{2}}$ and a temperature of 100 °C, the peaks exhibit negligible movement (see Figure 6b–d), suggesting minimal stress relaxation or defect recovery under these conditions. Similarly, under this initial EWF treatment conditions ($3300\;\frac{A}{mm^{2}}$ at 100 °C), only a slight modification in the FWHM values is observed. This indicates that under these conditions, the EWF treatment does not significantly reduce the dislocation density or relax the lattice strains induced by 50% rolling. Conversely, when the treatment involves a lower current density ($700\;\frac{A}{mm^{2}}$) but at a higher temperature (200 °C), the peaks shift rightward, approaching the positions observed in the pristine state. This indicates a substantial reduction in lattice strain and dislocation density, suggesting effective defect mitigation.

The rightward shift underlines the role of increased thermal energy in enhancing the mobility of dislocations and possibly facilitating the recrystallization process, thus restoring the lattice closer to its original, less strained condition. In addition, the treatment at $700\;\frac{A}{mm^{2}}$ and 200 °C results in a decrease in FWHM. Under these conditions, the FWHM values not only reverse the increases caused by rolling but also drop below those of the pristine material. This decrease in FWHM suggests a substantial reduction in defect density, surpassing even the initial state of the alloy, as confirmed by the complementary Electron Backscatter Diffraction (EBSD) analysis in the previous section. This decrease also signifies effective stress relief, likely due to the combined effects of elevated temperature enhancing atomic mobility and the mechanical impact of EWF facilitating dislocation movement and rearrangement.

**Figure 7:**
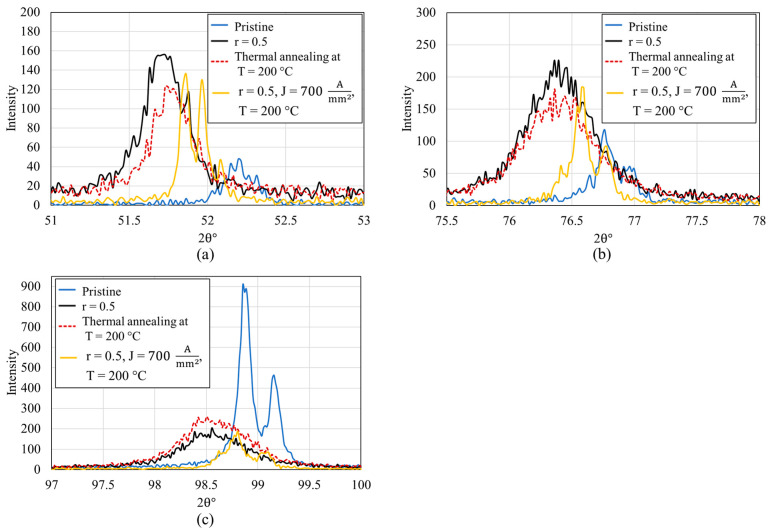
XRD patterns of thermal annealing and EWF-assisted annealing at 200 °C of 50% rolled sample. (**a**) peak A (**b**) peak B and (**c**) peak C as defined in Figure 6.

While EWF-assisted annealing at 200 °C demonstrated effective defect mitigation, evidenced by shifts in peak positions and reductions in FWHM, aligning them closer to the pristine state, the XRD analysis showed minimal shift in peak positions and no significant change in the FWHM during the thermal annealing process at 200 °C, as illustrated in Figure 7. Moreover, the XRD results in Figure 7 further support the earlier discussed EBSD findings, which indicated that LAGBs and Kernel Average Misorientation (KAM) values were unchanged by thermal annealing alone, as shown in Figure 6. This observation underscores the synergistic effect of thermal and athermal energies provided by EWF at elevated temperatures, enabling more efficient defect resolution even under severe deformation conditions. By optimizing both the current density and temperature, it is possible to tailor the microstructural recovery process, achieving effective defect mitigation at substantially lower temperatures than would be required with conventional thermal annealing alone.

### 3.4. Grain Coarsening Due to EWF Annealing

From the EBSD analysis, it is also observed that the pristine sample has a mean grain size of 6 μm. Post 50% rolling, the mean grain size increases to 7.6 μm, and after thermal annealing at 200 °C, the grain size was almost identical to 7.8 μm. However, the EWF-assisted annealing at 200 °C results in an increase in mean grain size to 12 μm.

**Figure 8:**
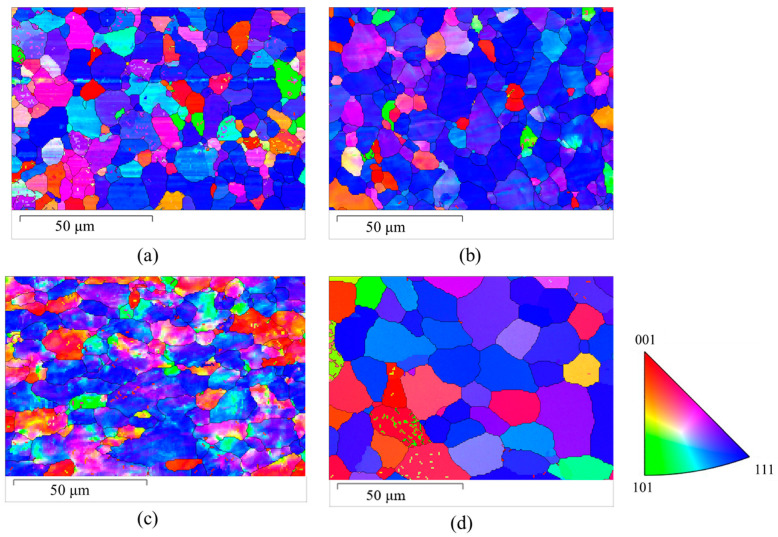
The Electron Backscatter Diffraction (EBSD) maps represent different stages of treatment for a FeCrAl alloy: (**a**) pristine (**b**) after being 50% rolled, (**c**) after thermal annealing at 200 °C, and (**d**) after undergoing EWF-assisted annealing at 200 °C.

The increase in grain size to 12 μm during EWF-assisted annealing at 200 °C, as shown in Figure 8, can be explained by the combined thermal and athermal effects unique to this process. Electron Wind Force (EWF) enhances atom mobility beyond what thermal energy alone can achieve, facilitating the movement and rearrangement of atoms within the crystal lattice. This process effectively reduces dislocation density, lowering the barriers to grain boundary migration and allowing grains to grow larger by absorbing neighboring smaller grains or shifting boundaries more freely. Additionally, the synergy between the elevated temperature and electron wind forces may increase grain boundary mobility and potentially trigger dynamic recrystallization. These factors together promote significant grain growth as residual stresses are relieved more effectively compared to traditional thermal annealing, leading to the observed coarsening of grains. This distinct outcome highlights the effectiveness of combining thermal and electron wind forces to achieve superior microstructural recovery compared to traditional thermal annealing.

## 4. Conclusions

This study demonstrated that Electron Wind Force (EWF) combined with thermal treatments significantly enhances defect mitigation in FeCrAl alloys subjected to different degrees of cold rolling. The traditional thermal annealing at 200 °C proved ineffective for alleviating defects, due to its inability to reach the recrystallization threshold of over 700 °C necessary for FeCrAl alloys. In contrast, EWF treatment at 100 °C with a current density of 3300 A/mm^2^ was effective in reducing defects for alloys rolled to 25%, but insufficient for those rolled to 50%. This discrepancy was attributed to the denser and more complex dislocation networks present in more heavily rolled samples, as analyzed through a simplified mathematical model quantifying dislocation climbing rates. Significantly, a combined approach utilizing both thermal and athermal energies at 200 °C with a lower current density of 700 A/mm^2^ markedly improved defect mitigation for the 50% rolled samples. Additionally, EWF-assisted annealing at 200 °C promoted substantial grain coarsening, with grain sizes increasing to 12 µm, in contrast to negligible changes observed with solely thermal annealing. These findings underscore the potential of synergistic EWF and thermal treatments as a more effective alternative to conventional methods for enhancing the microstructural properties of heavily deformed materials.

## Figures and Tables

**Table 1:** Comparison of LAGB concentrations to demonstrate the efficacy of treatment parameters across different experimental conditions. J is the current density, r = 0.25 and 0.5 represent 25% and 50% rolling, respectively.

Condition	LAGBs Concentration (%)
pristine	54.8
r = 0.25	82.4
r = 0.25, J = $3300\;\frac{A}{mm^{2}}$, T = 100 °C	47.5
r = 0.5	85.2
r = 0.5, J = $3300\;\frac{A}{mm^{2}}$, T = 100 °C	78.5
r = 0.5, J = $700\;\frac{A}{mm^{2}}$, T = 200 °C	10.1
r = 0.5, thermal annealing at T = 200 °C	81.4

**Table 2:** The 2θ° positions of three peaks under various conditions. J is the current density, r = 0.25 and 0.5 represent 25% and 50% rolling, respectively.

Peak	Pristine	r = 0.25	$r=0.25,\;J=3300\frac{A}{mm^{2}}$, T = 100 °C	r = 0.5	$r=0.5,\;J=3300\frac{A}{mm^{2}}$, T = 100 °C	$r=0.5,\;J=700\frac{A}{mm^{2}}$, T = 200 °C	Thermal Annealing at T = 200 °C
A	52.21	52.163	52.229	51.7	51.628	51.973	51.74
B	76.76	76.74	76.77	76.35	76.21	76.6	76.36
C	98.85	98.77	98.937	98.50	98.52	98.80	98.52

**Table 3:** Full width at half max (FWHM) of three peaks under various conditions in (°) unit. J is the current density, r = 0.25 and 0.5 represent 25% and 50% rolling, respectively.

Peak	Pristine	r = 0.25	$r=0.25,\;J=3300\frac{A}{mm^{2}}$, T = 100 °C	r = 0.5	$r=0.5,\;J=3300\frac{A}{mm^{2}}$, T = 100 °C	$r=0.5,\;J=700\frac{A}{mm^{2}}$, T = 200 °C	Thermal Annealing at T = 200 °C
A	0.2151	0.32	0.215	0.293	0.27	0.147	0.304
B	0.284	0.449	0.413	0.542	0.4	0.259	0.548
C	0.36	0.571	0.34	0.591	0.572	0.217	0.603

## Data Availability

The raw data supporting the conclusions of this article will be made available by the authors on request.
